# Small Bowel Adenocarcinoma as the Cause of Gastrointestinal Bleeding in Celiac Disease: A Rare Malignancy in a Common Disease

**DOI:** 10.1155/2015/865383

**Published:** 2015-07-28

**Authors:** Jaleh Fallah, Maxwell Eyram Afari, Alfredo C. Cordova, Adam J. Olszewski, Taro Minami

**Affiliations:** ^1^Department of Medicine, Memorial Hospital of Rhode Island, Pawtucket, RI 02860, USA; ^2^The Warren Alpert Medical School of Brown University, Pawtucket, RI 02860, USA; ^3^Department of Surgery, Memorial Hospital of Rhode Island, Pawtucket, RI 02860, USA; ^4^Division of Pulmonary, Critical Care and Sleep Medicine, Memorial Hospital of Rhode Island, Pawtucket, RI 02860, USA

## Abstract

*Introduction*. Celiac disease is associated with an increased risk of small bowel malignancies, particularly lymphoma. Its association with small bowel carcinoma is less known. *Case Description*. We report a case of an 89-year-old woman with celiac disease who experienced recurrent episodes of gastrointestinal bleeding and was ultimately found to have adenocarcinoma of the small intestine. *Discussion and Evaluation*. Diagnosis of small bowel adenocarcinoma is often delayed because of the need for specialized modalities, which are often deferred in the inpatient setting. Although resection is the modality of choice for small bowel tumors, a majority is either locally advanced or metastatic at diagnosis, and even localized cancers have worse prognosis than stage-matched colorectal tumors. The role of adjuvant chemotherapy is uncertain, but it is often offered extrapolating data from other gastrointestinal cancers. Small bowel carcinomas occurring in the context of celiac disease appear to be associated with higher rates of microsatellite instability than sporadic tumors, although other specific genomic abnormalities and mechanisms of carcinogenesis in celiac disease remain unknown. *Conclusion*. Recurrent episodes of gastrointestinal bleeding in a patient with celiac disease should prompt an early evaluation of the small bowel to assure timely diagnosis of carcinoma at an early curable stage.

## 1. Introduction

Celiac disease is a common disease, with a prevalence of approximately 1-2% in North and South America, North Africa, India, and the Middle East [1]. Iron deficiency is a common clinical feature of this disease and is related to malabsorption rather than gastrointestinal blood loss. Celiac disease is known to be associated with an increased risk of malignancy, particularly lymphoma [2, 3]. However, less is known about the risk of gastrointestinal carcinoma associated with celiac disease. We have encountered a case of adenocarcinoma of the small intestine with celiac disease, a rare malignancy in a common disease.

## 2. Case Presentation

An 89-year-old woman with a history of celiac disease, prior gastrointestinal bleeding, and dementia was admitted to our Intensive Care Unit for a recurrent gastrointestinal bleeding. The celiac disease had been diagnosed two years earlier by an endoscopic biopsy of the duodenum (demonstrating a subtotal villous atrophy) and with a correlative serum tissue transglutaminase IgA antibody (162 units, reference range 0–19 units). The patient had not been compliant with the recommended gluten-free diet because of early dementia and was treated with additional oral budesonide because of chronic abdominal discomfort. She was in her usual state of health until two days prior to this admission when she developed nausea and had multiple bouts of coffee ground emesis, with which she presented to the emergency room. She reported poor appetite and significant weight loss during the prior year, which had been ascribed to celiac disease-related malabsorption and led to therapy with oral budesonide. Her medical history was additionally significant for prior admission with similar symptoms of nausea, vomiting, and gastrointestinal bleeding with severe anemia three, six, and twelve months prior to the index presentation. Esophagogastroduodenoscopy (EGD) had been performed on all these occasions, showing gastritis and duodenitis with biopsies consistent with celiac disease. There was no history of tobacco or alcohol use and no family history of celiac disease or malignancies.

**Figure 1:**
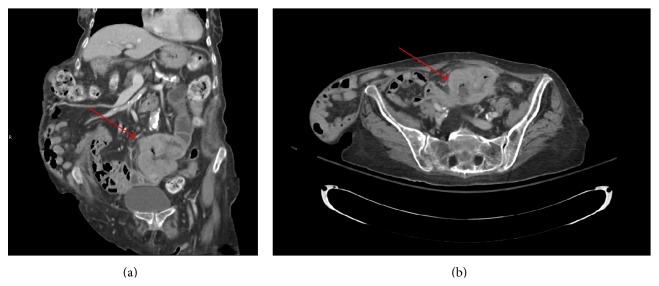
Axial (a) and coronal (b) projections of the computerized tomography of the abdomen. Arrows point to the small bowel tumor.

On admission, the patient was hypotensive with blood pressure of 93/49 mm Hg and heart rate of 86 beats per minute and afebrile and her respiratory rate was 18 breaths per minute. Physical examination revealed a palpable left paraumbilical mass and a 20 cm × 20 cm reducible hernia in the right abdominal wall. Laboratory work-up revealed hemoglobin of 6.9 g/dL (reference range, 12.0–16.0 g/dL). Serum chemistry, liver function tests, electrocardiogram, and chest radiograph were within normal limits. Abdominal computed tomography (CT) with intravenous contrast revealed a proximal dilatation of an abnormally thickened segment of the small bowel suspicious for a malignancy (Figure 1). The patient was resuscitated with intravenous fluids and a red cell transfusion.

**Figure 2:**
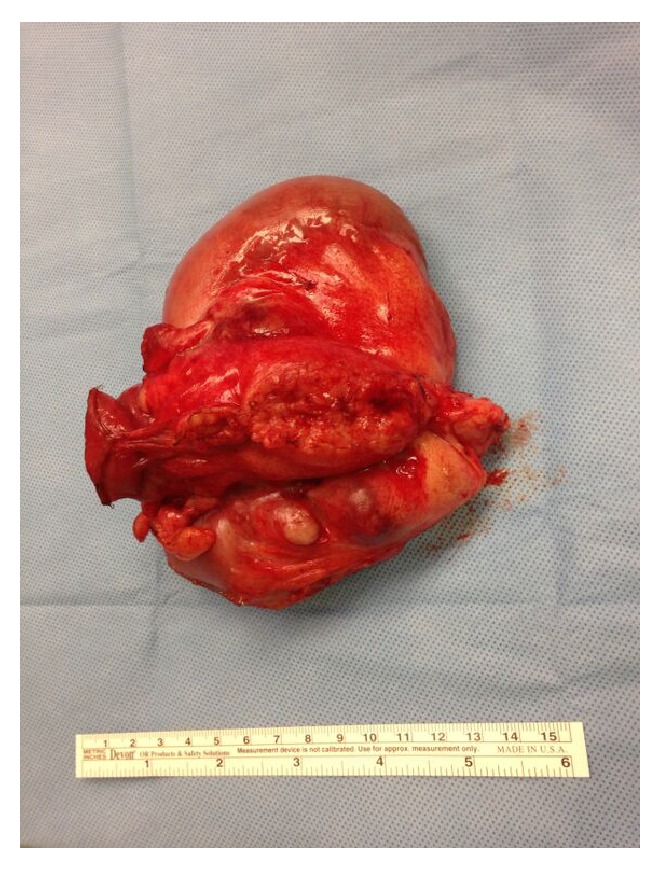
Photograph of the gross resection specimen of the small bowel segment containing adenocarcinoma.

**Figure 3:**
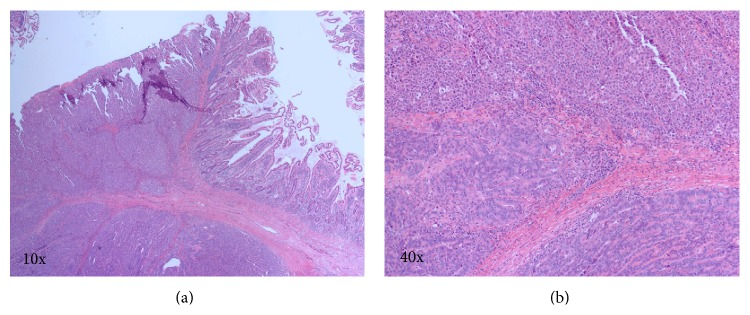
Microscopic images of the tumor from the histopathologic specimen, hematoxylin and eosin stain. (a) Low-power magnification (10x) shows normal small bowel mucosa (right) and invasive adenocarcinoma (left). (b) High-power magnification (40x) shows two patterns of differentiation, with poorly differentiated carcinoma in the upper part of the panel.

On third admission day, after hemodynamic stability was established, she underwent laparotomy. A large (15 × 10 cm) small bowel tumor located approximately 20 cm from the ligament of Treitz was resected (Figure 2). There was no evidence of metastatic lesions in the peritoneum or liver during intraoperative inspection of all quadrants of the abdominal cavity. Pathologic examination revealed a poorly differentiated adenocarcinoma (final tumor size 6.5 cm) with invasion into an adjacent portion of the small intestine and to the serosal surface, with metastases to 4 out of 12 resected lymph nodes, staged as pT4b pN2 M0 or stage III according to the American Joint Commission on Cancer staging system (Figure 3). The tumor cells were positive for immunohistochemical markers of adenocarcinoma (CDX2 and CK7) and negative for CK20, vimentin, neuroendocrine markers (synaptophysin, chromogranin, and CD56), and lymphoid antigens (CD3, CD20, and CD45). After the surgery, the patient elected palliative care and did not receive any further cancer-directed therapy.

## 3. Discussion

Small bowel carcinoma is a rare clinical entity with an annual incidence of 0.66 cases per 100,000 person-years in the United States, which is higher for men (0.80 per 100,000) than for women (0.55) [4]. Neuroendocrine tumors (39%, mostly carcinoids) are more common than carcinomas (31%), while less common histologies encompass lymphomas (18%) and sarcomas (10%). Although the small intestine is three times longer than the large intestine, it is the primary location for only 5% of all gastrointestinal malignancies [5].

Despite the fact that lower gastrointestinal bleeding is a common cause of hospitalization in the elderly, the diagnosis can be challenging and the diagnostic differential is broad. In a study of 1,112 patients admitted to an urban emergency medical center, diverticulosis (33.5%), hemorrhoids (22.5%), and carcinoma (12.7%) were the most common etiologies [6]. In general the causes of gastrointestinal bleeding can be grouped into anatomic (diverticular disease), vascular (hemorrhoid, ischemic colitis, and angiodysplasia), neoplastic, and inflammatory (infectious colitis, inflammatory bowel disease) causes.

The diagnosis of small bowel carcinoma is difficult and often delayed. Clinical presentation is typically nonspecific with symptoms that include nausea, vomiting, weight loss, abdominal pain, and anemia. In our patient, the diagnostic process was further complicated by presence of a known small bowel disease (celiac disease) commonly associated with iron deficiency anemia, negative endoscopy, and colonoscopy. Iron deficiency anemia is present in 46% of patients with celiac disease and is typically caused by malabsorption of iron in the proximal small intestine and usually resolves with strict adherence to gluten-free diet and oral iron supplementation, although it may be refractory in up to 20% [7]. In our case, the correct diagnosis for the underlying cause of recurrent anemia was only made during the fourth hospital admission.

Serum cancer markers carcinoembryonic antigen (CEA) and Ca19.9 may be elevated in cases of small bowel carcinoma but are neither sensitive nor specific and should not be used for diagnostic purposes. Even in metastatic disease CEA is elevated only in 30% and Ca19.9 in 41% of cases [8]. Extrapolating the established guidelines for colorectal carcinoma, these markers, if elevated at diagnosis, can be used for surveillance after surgery or for assessment of disease activity in patients with metastatic cancer [9]. Values of CEA or Ca19.9 were not obtained in our patient who elected palliative care only after her cancer diagnosis.

Because small bowel is not accessible during standard endoscopic procedures performed in the setting of gastrointestinal bleeding, a high index of suspicion is required to identify pathology in this segment of the gastrointestinal tract. Clinical examination has a low sensitivity for small bowel tumors until they become palpable, as it was in our case. CT may provide suggestive information about nodal, extramucosal involvement and distant metastases. However, it can miss superficial lesions and presence of overlapping bowel loops may lead to false negative diagnosis. The addition of enterography to CT circumvents these problems and increases the sensitivity [10]. Magnetic resonance imaging (MRI) is rapidly gaining clinical utility in the evaluation of small bowel disease. It advantageously avoids ionizing radiation, can assess bowel peristalsis, and provides a higher resolution, but it has its own challenges such as cost and accessibility. CT is better in the assessment of a perforation or bowel obstruction and concomitantly helps in directing interventional therapies [10].

Capsule endoscopy may identify a site of occult bleeding or malignancy but poses a risk of capsule retention when there is an impending obstruction [11]. Double balloon enteroscopy overcomes the above limitation and has the advantage of ability to obtain biopsy or providing endoscopic therapy [12]. These newer diagnostic techniques can expedite the diagnosis, but in clinical practice they are often deferred to the outpatient setting in patients presenting with acute gastrointestinal bleeding. Our case illustrates the importance of pursuing such evaluations when repeated episodes of unexplained gastrointestinal bleeding occur in order to avoid delay in the diagnosis of a potentially curable malignancy.

The pathogenesis of small bowel adenocarcinoma is not entirely known due to the rarity of the disease. Genetic syndromes like Peutz-Jeghers (relative risk, 520), hereditary nonpolyposis colon cancer (relative risk, 103 to 291 depending on specific mutation), and familial adenomatous polyposis (relative risk, 330) are risk factors for small bowel adenocarcinoma [13]. Conditions associated with chronic inflammation of the small intestine are also major risk factors for this malignancy and include Crohn's disease and celiac disease. Thirteen percent of small bowel adenocarcinomas have been shown to be associated with celiac disease [14]. Cases of small bowel adenocarcinoma in patients with celiac disease have been previously reported [15–17].

Celiac disease is an autoimmune disorder which results from the interplay of immune, genetic, and environmental factors. The lymphocytic infiltrate could potentially induce an immunological response disrupting the epithelium and causing premalignant changes. In a series of 94 cases of small bowel adenocarcinoma from the Mayo Clinic, evidence of flat dysplasia at the tumor margin was noted in 50% of cases associated with celiac disease [18]. Although some authors suggested that carcinomas in celiac disease may arise from adenomatous polyp [19], others found no increased incidence of duodenal adenomas in celiac disease [20].

Microsatellite instability, a hallmark of the hereditary nonpolyposis colon cancer (Lynch) syndrome, is a molecular feature commonly encountered in small bowel adenocarcinoma, where it is present in about 20–40% of cases [21–23]. Several studies have demonstrated particularly high prevalence of microsatellite instability (70–80%) in tumors associated with celiac disease [24, 25]. A comparative genomic hybridization study of 15 celiac disease-related and 33 unrelated small bowel adenocarcinomas showed similar chromosomal aberrations between the two subsets, but there was a higher rate of* APC* gene promoter hypermethylation and of microsatellite instability in celiac disease-related tumors (67% versus 33%) [26].

Small bowel carcinogenesis in Crohn's disease differs from celiac disease. Cytokines, released due to the chronic inflammatory state, interact with cell surface receptors and target genes thus promoting carcinogenesis [27]. Unlike in celiac disease where proximal jejunum predominates as the location of adenocarcinomas, Crohn's disease affects mostly the distal ileum.

In the absence of randomized trials, the optimal treatment for small bowel carcinoma is still uncertain and is essentially extrapolated from the clinical experience in colorectal cancer. Surgery remains the treatment of choice in the absence of metastatic disease and is often required even in the metastatic setting due to high likelihood of obstruction or severe hemorrhage [28]. Because most patients are diagnosed with advanced disease and have high-grade histology [4], small bowel adenocarcinoma generally carries a poor prognosis with 5-year relative survival of 28% without surgery and 32% after resection [29]. Adjuvant chemotherapy using fluorouracil-based regimens approved in colorectal cancer is commonly offered for patients with stage II or III disease [30]. It may also provide benefits in selected patients with metastatic disease, where a 27% response rate, median progression-free survival of 6.6 months and median overall survival of 15 months, was reported among patients receiving palliative chemotherapy [8]. In one phase II trial of the combination of capecitabine and oxaliplatin, a response rate of 50% was observed, with median time to progression of 11.3 months and median overall survival of 20.4 months [31]. It is important however to recognize that patients with significant comorbidities or poor performance status may not derive any benefit from adjuvant or palliative chemotherapy, and, in those cases, as it was in ours, supportive care alone may be appropriate.

Identification of molecular pathways involved in the pathogenesis of small bowel cancer holds a promise of developing more efficacious therapies. A screening assay of 140 compounds suggested utility of the halichondrin B analogue eribulin in cases with* Wnt-β-catenin* pathway mutation present in 20% of cases [21]. A multiplex assay analysis of 83 cases showed frequent mutations in* KRAS* (43%),* TP53* (41%),* PIK3CA* (8%),* BRAF* (6%), and* ERBB2* (8%) genes, for which targeted agents are available or currently studied [32].

## 4. Conclusion

When patients with celiac disease present with gastrointestinal bleeding, small intestinal malignancy has to be included in differential diagnosis if endoscopy and colonoscopy do not reveal an obvious source of blood loss. Early evaluation for a malignancy using a high-yield technique should be considered to avoid a diagnostic delay and the poor prognosis associated with advanced cancers.

## Abbreviations

CT: Computerized tomography
EGD: Esophagogastroduodenoscopy.
